# Glioma Cells Acquire Stem-like Characters by Extrinsic Ribosome Stimuli

**DOI:** 10.3390/cells10112970

**Published:** 2021-11-01

**Authors:** Yuki Shirakawa, Kunimasa Ohta, Shunsuke Miyake, Ayumi Kanemaru, Akari Kuwano, Kou Yonemaru, Shota Uchino, Michiko Yamaoka, Yuki Ito, Naofumi Ito, Takuichiro Hide, Naoki Shinojima, Akitake Mukasa, Hideyuki Saito, Hirofumi Jono

**Affiliations:** 1 Department of Clinical Pharmaceutical Sciences, Graduate School of Pharmaceutical Sciences, Kumamoto University, Kumamoto 860-8556, Japan; y.shirakawa.da@juntendo.ac.jp (Y.S.); 191y2005@st.kumamoto-u.ac.jp (S.M.); 197y2002@st.kumamoto-u.ac.jp (A.K.); ak.1212@icloud.com (A.K.); 179p1054@st.kumamoto-u.ac.jp (K.Y.); 175p1008@st.kumamoto-u.ac.jp (S.U.); yfe31628@gmail.com (M.Y.); 161p1003@st.kumamoto-u.ac.jp (Y.I.); saitohide@kuh.kumamoto-u.ac.jp (H.S.); 2 Department of Stem Cell Biology, Faculty of Arts and Science, Kyushu University, Fukuoka 819-0385, Japan; ohta9203@artsci.kyushu-u.ac.jp; 3 Department of Pharmacy, Kumamoto University Hospital, Kumamoto 860-8556, Japan; 4 Department of Developmental Neurobiology, Graduate School of Life Sciences, Kumamoto University, Kumamoto 860-8556, Japan; mikan03orange@gmail.com; 5 Department of Neurosurgery, Kitasato University School of Medicine, Sagamihara 252-0375, Japan; thide@med.kitasato-u.ac.jp; 6 Department of Neurosurgery, Graduate School of Medical Sciences, Kumamoto University, Kumamoto 860-8556, Japan; nshinojima-kuh@umin.ac.jp (N.S.); mukasa@kumamoto-u.ac.jp (A.M.)

**Keywords:** glioblastoma, ribosome, ribosomal protein S6, ribosome-induced cancer cell spheroid

## Abstract

Although glioblastoma (GBM) stem-like cells (GSCs), which retain chemo-radio resistance and recurrence, are key prognostic factors in GBM patients, the molecular mechanisms of GSC development are largely unknown. Recently, several studies revealed that extrinsic ribosome incorporation into somatic cells resulted in stem cell properties and served as a key trigger and factor for the cell reprogramming process. In this study, we aimed to investigate the mechanisms underlying GSCs development by focusing on extrinsic ribosome incorporation into GBM cells. Ribosome-induced cancer cell spheroid (RICCS) formation was significantly upregulated by ribosome incorporation. RICCS showed the stem-like cell characters (number of cell spheroid, stem cell markers, and ability for trans differentiation towards adipocytes and osteocytes). In RICCS, the phosphorylation and protein expression of ribosomal protein S6 (RPS6), an intrinsic ribosomal protein, and STAT3 phosphorylation were upregulated, and involved in the regulation of cell spheroid formation. Consistent with those results, glioma-derived extrinsic ribosome also promoted GBM-RICCS formation through intrinsic RPS6 phosphorylation. Moreover, in glioma patients, RPS6 phosphorylation was dominantly observed in high-grade glioma tissues, and predominantly upregulated in GSCs niches, such as the perinecrosis niche and perivascular niche. Those results indicate the potential biological and clinical significance of extrinsic ribosomal proteins in GSC development.

## 1. Introduction

Glioblastoma multiform (GBM) is the most common malignant brain tumor [1]. Despite the existence of several treatments, such as chemo-radio therapy and surgical resection, the median survival of patients is about 1 year [2], and several standard therapeutic strategies have not improved over the past three decades. GBM frequently shows characteristics of chemo-radio resistance and recurrence after resection [3]. Part of the reason for chemo-radio resistance and recurrence is the presence of a small population of stem cell character cells called GBM stem-like cells (GSCs) [3,4,5]. Moreover, since GSCs also play important roles in angiogenesis, self-renewal, unlimited proliferation maintains tumor tissue of GBM, and low mitotic activity, those characteristics protect GSCs from therapeutic approaches directed against actively dividing cells [5,6]. Although recent studies have revealed that GSCs predominantly localize GSC niches, such as the necrosis niche, peri-vascular niche, and border niche [7,8], the mechanisms of the development of stem-like characters are largely unknown. Therefore, to remedy the survival time of patients, it is quite important to reveal the mechanism underlying GSCs’ development and to identify the key target molecule for new GBM therapeutic strategy by targeted GSCs.

Ribosomes, as proteins that manufacture organelles in the cell, regulate the general functions of numerous biological phenomena via protein synthesis [9]. Ribosomes comprise small and large subunits [10], and contain approximately 80 ribosomal proteins, which regulate tumorigenesis, development, immune response, and several diseases [10,11]. Of particular interest, recent studies have reported that the incorporation of ribosomal proteins into somatic cells promotes reprogramming and lineage transdifferentiation toward multipotency [12,13]. These cells indeed differentiate into germ layer-derived cells upon differentiation, such as adipocytes, osteocytes, and chondrocytes [13,14], suggesting that ribosome proteins may have the potential to play pivotal roles in reprogramming and development of stem cell characters. Ito et al. reported that ribosome incorporation into somatic cells depends on trypsin-activated endocytosis and ribosomes may interact with several intrinsic cell signals. Meanwhile, our recent studies revealed that ribosomal proteins are overexpressed at border niches in GBM tissues [15]. A recent study also showed that translation of ribosomal RNA and nucleolar regulation promoted glioma tumorigenesis, such as stem cell characters [16]. Furthermore, we also revealed that ribosome including RPS6 played crucial roles in stem-like characters in glioma cells in GBM patients [15]. However, the relationship between GSC development and ribosome incorporation as a key trigger for reprogramming is totally unknown.

In this study, to reveal the mechanism underlying GSC development in GBM tissues, we focused on the clinical and biological significance of ribosome proteins and investigated whether the incorporation of ribosome into GBM cells triggers stem-like characters and mechanisms of promoting stemness in GBM cells.

## 2. Materials and Methods

### 2.1. Antibodies and Reagents

Rabbit polyclonal anti-RPS6 antibody and rabbit polyclonal anti-pRPS6 (Ser235/236) antibody were obtained from Abcam (Cambridge, MA, USA). Mouse polyclonal anti-β-actin antibody, rabbit polyclonal anti-STAT3 antibody, rabbit polyclonal anti-p-STAT3(Tyr705) antibody, and rabbit polyclonal anti-SOX2 antibody were purchased from CST (Danvers, MA, USA). Mouse monoclonal anti-CD34 was purchased from Leica (Wetzlar, Germany). Mouse monoclonal anti-Nestin antibody was obtained from Merck (Darmstadt, Germany). PF4708671 was obtained from Selleck (Houston, TX, USA) and dissolved in 5 µM DMSO (Sigma-Aldrich, St. Louis, MO, USA) and then diluted in culture medium.

### 2.2. Cell Line and Cell Culture

The human GBM cell line (U251MG cells) was obtained from the Japanese Collection of Research Bio Resources Cell Bank (Ibaraki City, Osaka, Japan). U251MG cells were cultured in Dulbecco’s modified Eagle’s medium and Ham’s medium (Gibco) with 10% FBS in an atmosphere containing 5% CO_2_ in air at 37 °C.

### 2.3. Preparation of Ribosomes

Prokaryotic ribosomes were purified by ultracentrifugation, as previously described [16]. Briefly, 1% volume of pre-cultured solutions (stationary-phase bacteria) were inoculated into fresh culture medium, and incubated until the bacteria reached the late-stationary phase. The cultured bacteria were immediately chilled, collected by centrifugation, and washed with TMA-I buffer [10 mM Tris-HCl (pH 7.8), 30 mM NH4Cl, 10 mM MgCl2, and 6 mM 2-mercaptoethanol]. All operations were further performed at 4 °C. The collected bacteria were suspended in TMA-I buffer and disrupted by sonication. The debris of bacteria was removed by centrifuging the suspension at 10,000× *g*, and the supernatant was collected and centrifuged at 158,000× *g* for 30 min. The obtained supernatant was collected and centrifuged at 116,000× *g* for 6 h. The sediment was dissolved in 1 mL of TMA-I and layered on 30% sucrose containing TMA-I, and centrifuged at 158,000× *g* for 15 h. The sucrose-cushion-purified 70 S ribosome precipitate in TMA-I supplemented with 10% glycerol was diluted into PBS and chilled rapidly in liquid nitrogen. The ribosome samples were stored at −80 °C.

To purify human GBM ribosomes, ultracentrifugation was performed as reported previously [12]. Human GBM cell line (U251MG cells, 4 × 10^6^ cells) pellets were suspended in HMK buffer [20 mM HEPES (pH 7.4), 100 mM KOAc, 7.5 mM Mg(OAc)_2_, 1 mM DTT, and 0.5 mM phenylmethyl sulfonyl fluoride] and disrupted by vigorous shaking with glass beads (FastPrep; MP Bio Japan K.K., Tokyo, Japan). Cell debris of GBM and glass beads (WAKO) was removed by centrifugation and the supernatant was filtered through a 0.45-µm filter cassette. The crude GBM cell lysate was centrifuged on a high-salt sucrose cushion (30% sucrose, 500 mM KOAc, 25 mM Mg(OAc)2, 1 mM DTT, and 0.5 mM phenylmethylsulfonyl fluoride) at 355,040× *g* (*CP80NX*, HITACHI, Tokyo, Japan) for 60 min. The sucrose-cushion-purified precipitate was dissolved by HMK buffer, aliquoted, and chilled in liquid nitrogen. The GBM ribosome samples were stored at −80 °C.

### 2.4. Ribosome-Induced Cell Cluster Formation

Previous studies reported that the incorporation of ribosome into somatic cells induced stem-like characters [14]. For buffer desalting, ribosome dilution buffer was replaced by PBS through ultrafiltration (Amicon Ultra 3000 Da MW; Merck, Darmstadt, Germany). Cells were collected by conventional trypsin digestion. The obtained cell suspensions were adjusted to approximately 1 × 10^5^ cells/500 µL and then applied to a 24-well plate. Ribosome solution (10 μg) was added on the culturing plate surface and spread equally into the well by using a micro-pipette. The cells (U251MG) were cultured on the plate with purified eukaryotic/prokaryotic ribosomes. The plated cells were cultured for 7 days, with half of the medium replaced every 3–4 days. All immunocytochemistry, expression, and fate-conversion experiments were performed using ribosome incorporation cells that were cultured for 14 days in DMEM/F12 at 5% CO_2_ 37 °C.

### 2.5. Sphere Formation Assay

U251MG cells (10^4^ cells/100 µL/well) were cultured in DMEM/F12 (https://www.thermofisher.com/jp/en/home/technical-resources/media-formulation.54.html: Gibco accessed on 1 November 2021) culturing condition (serum-free medium as recently reported [14]) using a 96-well plate (Corning Incorporated, Corning, NY, USA) for 72 and 120 h. The spheres (size > 50 µm) were enumerated by using a microscope.

### 2.6. Western Blot Analysis

U251MG cells (10^5^ cells/1000 µL/well) were cultured in DMEM/F12 culturing condition (serum-free medium as recently described [14]) using a 24-well plate (Corning) for 72 h. U251MG Cells were lysed in ice-cold lysis buffer containing phosphatase inhibitor (CST). Whole proteins from cultured cells were extracted using cell lysis buffer following the manufacturer’s instructions (CST). Proteins were separated by SDS-PAGE in a 10% resolving gel and electro-transferred onto polyvinylidene difluoride membranes (Merck). Membranes were blocked in 3% skim milk (GE Healthcare, Minato-ku, Tokyo, Japan) in TBS-T at ambient temperature for 1 h with agitation, and incubated with the following primary antibodies overnight at 4 °C: rabbit polyclonal anti-RPS6 antibody (1:1000), mouse polyclonal anti-β-actin antibody (1:1000), rabbit polyclonal anti-STAT3 (1:1000), rabbit polyclonal anti-p-STAT3 (Tyr705) (1:1000), rabbit polyclonal anti-SOX2 (1:1000), and mouse monoclonal anti-Nestin (1:1000) antibodies. After three washes, the membranes were incubated with horseradish peroxidase-conjugated secondary antibody (rabbit: GE Healthcare, mouse: GE Healthcare) with 3% skim milk for 1 h. Finally, immunoreactive protein bands were visualized by using ECL select detection reagents (GE Healthcare) and captured using the LAS4000EPUVmini (GE Healthcare).

### 2.7. Differentiation into Adipocytes and Osteocytes

U251MG cells were trypsinized and 1 × 10^5^ cells were suspended in 1 mL of DMEM/F12. Half of the medium was replaced every 3 days. One set of cells was cultured for up to 2 weeks (D14). For transdifferentiation, one or two RICCSs were collected from D14 culture and suspended in the specific differentiation induction medium. Adipocyte Differentiation Basal medium StemPro^®^ (Gibco, Rockford, IL, USA) and Osteoblast Differentiation Basal Medium StemPro^®^ (Gibco) were used to differentiate the RICCS into adipocytes and osteocytes, respectively. After 2 weeks of culture in the differentiation medium, the differentiated adipocytes were stained with Oil red O (SIGMA-ALDRICH) and osteoblasts were stained with with Alizarin Red S (SIGMA-ALDRICH).

### 2.8. Patients, Tissue Specimens, and Immunohistochemistry

For immunohistochemical analysis, glioma tissues were obtained from 10 patients (Grade 1: 3 cases, Grade 2: 2 cases, Grade 3: 2 cases, Grade 4: 3 cases) at Kumamoto University Hospital, Japan, after obtaining informed consent from the patients and in accordance with the guidelines of the Research Ethics Committee (Kumamoto University Hospital No. 1470). All tissue specimens were rapidly frozen after surgery and stored at −80 °C. Glioma patient tissues were fixed by formalin overnight, embedded in paraffin, and then cut into 3-μm-thick sections. The following antibodies were used to detect anti-gens: rabbit anti-pRPS6 (1:100; Abcam), rabbit anti-Nestin (1:200; Merck), and mouse anti-CD34 (1:100; Leica). Immunohistochemical reactions were visualized using a diaminobenzidine substrate system (Nichirei chuo-ku, Tokyo, Japan).

### 2.9. Double Immune Histochemistry Staining

For double immunofluorescence histochemical studies, resected GBM tissue was harvested from patients at Kumamoto University Hospital, Japan (research Ethics Committee Kumamoto University Hospital No. 1470). After GBM, patient tissue was fixed in formalin overnight, embedded in paraffin, and then cut into 3-μm-thick sections. The following antibodies were used to detect anti-gens: rabbit anti-pRPS6 (1:100; Abcam), rabbit anti-Nestin (1:200; Merck), and mouse anti-CD34 (Leica 1:100). Reactions were visualized by using a 2nd antibody rabbit-alexa594 and mouse-alexa488 (Thermo Fisher Scientific, Waltham, MA, USA). Nucleo was visualized by DAPI (Thermo Fisher Scientific). Fluorescence images were captured using a BZ-X700 (KEYENCE, Osaka, Japan).

### 2.10. Statistical Analysis

Data are expressed as mean ± standard deviation values. Student’s *t*-test was used to assess differences between the two groups. A *p*-value of less than 0.05 (* *p* < 0.05) was considered statistically significant.

## 3. Results

### 3.1. Incorporation of Prokaryotic Ribosome into U251MG Cells

**Figure 1:**
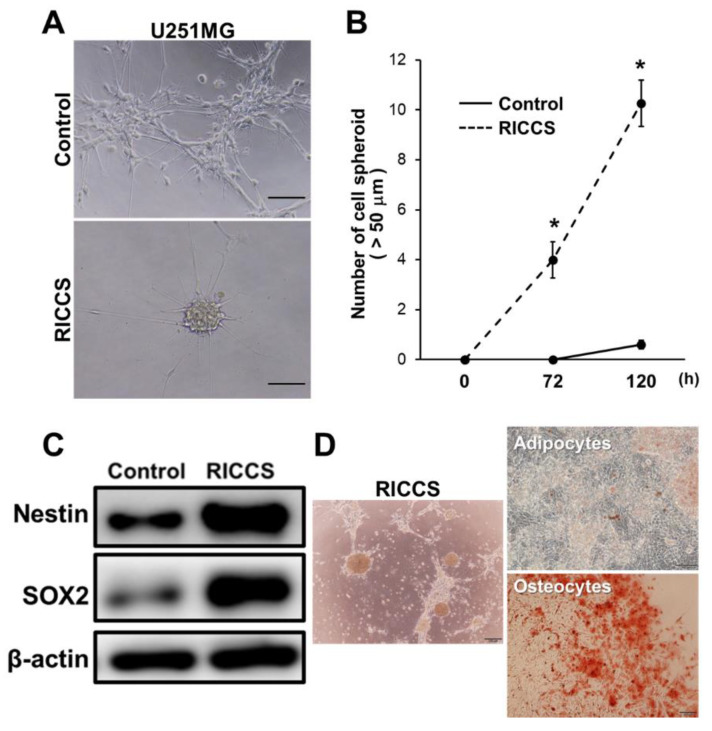
Ribosome incorporation into glioma cells induced stem cell characters. (**A**), Control (PBS) or ribosome-induced cancer cell spheroid (RICCS) in GBM (U251MG) cells. (**B**), Stem cell character was confirmed by sphere formation, and the graph showing the number of spheres (>50 µm) cultured for 0, 72, and 120 h. (**C**), Western blotting showing the stem cell markers, Nestin and SOX2 in RICCS. (**D**), Transdifferentiation of RICCS into adipocytes and osteocytes. RICCS from U251MG cells (let panel) and adipocyte and osteocyte transdifferentiation staining (right panel). Scale bar: 200 µm. * *p* < 0.05.

Several studies recently revealed that the incorporation of lactic acid bacteria induced stem cell properties in human dermal fibroblast (HDF) cells, and ribosomes represented an actual component for the induction of stem cell characters [13,17]. Although these studies suggested that ribosome incorporation induced stem cell characters in eukaryotic cells including human cells, the biological and clinical significance of ribosome incorporation was totally unclear. In this study, based on our study focusing on stem-like characters in glioma cells, we sought to elucidate the biological and clinical significance of the incorporation of ribosomes into glioma cells. As shown in Figure 1A, we first performed prokaryotic ribosome incorporation into the glioma cell line (U251MG), and revealed that ribosome incorporation indeed induced cell cluster formation. Ribosome-induced cancer cell spheroid (RICCS) formation was significantly upregulated by ribosome incorporation, compared with the control sample (Cont) (Figure 1B). The ribosome incorporation was confirmed by immunohistochemical staining for His-tag conjugated ribosome (Supplementary Figure S1). Moreover, stem cell markers, such as, SOX2, and Nestin, were upregulated in RICCS, compared with Cont (Figure 1C), suggesting that RICCS might exhibit stem-like characters in glioma cells. Furthermore, to evaluate the stem-like characters of RICCS, we assessed whether RICCS could transdifferentiate to some cell types. As shown in Figure 1D, RICCS indeed exhibited the potential to differentiate into adipocytes and osteocytes. These results indicate that prokaryotic ribosome incorporation induced stem cell characters in glioma cells, and suggest that extrinsic ribosome incorporation might play an important role in the biological function of glioma cells. 

### 3.2. Roles of Ribosomal Protein S6 in RICCS Formation

**Figure 2:**
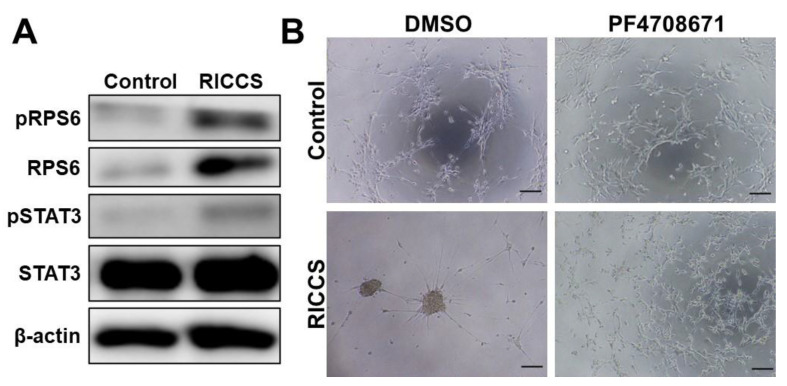
Involvement of intrinsic ribosomal protein S6 in RICCS formation. (**A**), Western blot showing that RPS6 phosphorylation, RPS6 expression, and STAT3 phosphorylation were induced in RICCS. (**B**), PF4708671 (RPS6 kinase inhibitor) treatment completely suppressed the sphere-forming ability in RICCS. Scale bar: 200 µm.

Of several ribosome proteins, ribosomal protein S6 (RPS6), a component of the 40S ribosomal subunit, plays important roles in cell proliferation and DNA repair [18]. Recent studies have revealed that hyperphosphorylation of RPS6 and RPS6 expression may predict malignant phenomena in several cancers, such as non-small cell lung cancer and leukemia, etc. [19,20]. More importantly, our recent study revealed that intrinsic RPS6 promotes stem-like characters in glioma cells [15]. Therefore, we next sought to determine the involvement of intrinsic RPS6 in RICCS formation. As shown in Figure 2A, extrinsic ribosome incorporation induced both expression and phosphorylation of intrinsic RPS6 in U251MG cells. In addition, STAT3 phosphorylation, important for cell signal activation in the development of GSCs [21,22,23,24], was also induced in RICCS (Figure 2A). Since the hyper-phosphorylation of RPS6 was observed with increased expression, we next evaluated the involvement of RPS6 phosphorylation in RICCS formation. As shown in Figure 2B, RICCS formation was significantly suppressed by RPS6 kinase inhibitor (PF47018671). No spheroid formation (>50 µm) was observed after the PF47018671 treatment (data not shown). PF4708671 decreased Nestin expression (Supplementary Figure S2). These findings suggest that extrinsic ribosome might induce stem cell characters in glioma cells through intrinsic RPS6 phosphorylation.

### 3.3. Effect of Glioma-Derived Ribosome on Stem Cell Characters in Glioma Cells

**Figure 3:**
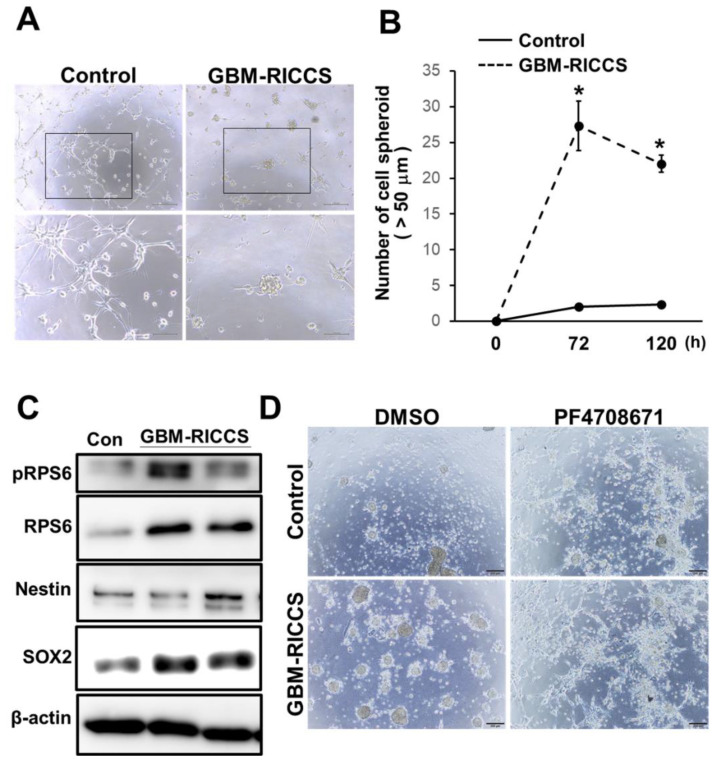
Effect of glioma-derived ribosome on stem cell characters in glioma cells. (**A**), Sphere-forming ability in GBM-RICCS induced by the incorporation of GBM ribosome or control (PBS). Bar: 100 µm (each down-scale picture), 200 µm (up-scale picture). (**B**), Stem cell characters were evaluated by sphere formation and the graph showing the number of spheres (>50 µm) cultured for 0, 72, and 120 h. (**C**), Western blotting showing the RPS6, pRPS6, and stem cell markers (Nestin and SOX2) in GBM-RICCS. (**D**), PF4708671 (RPS6 kinase inhibitor) treatment suppressed sphere-forming ability. Bar: 200 µm. * *p* < 0.05.

Although our results showed that extrinsic ribosome might induce stem cell characters in glioma cells through intrinsic RPS6 phosphorylation, the biological and clinical significance of the incorporation of ribosome into glioma cells are still unknown. As stated above, it is documented that GBM is frequently resistance to standard therapies and demonstrates recurrence, and stem cell characters regulate malignant phenomena in GBM patients [3]. However, the mechanisms promoting stem-like characters in GBM tissues are totally unknown. It should be noted that necrosis frequently occurs in glioma, including GBM patients. Our previous study further revealed that considerable amounts of ribosomal proteins exist in predominantly glioma stem-like cells, such as the peri-necrosis area, border area, pseudo-palisading area, and perivascular area [15]. Furthermore, necrosis burst cells and release glioma cell contents including RNA, DNA, and proteins [25]. Thus, in view of the biological and clinical significance of the incorporation of ribosome into glioma cells, we next focused on the effect of glioma-derived extrinsic ribosome in stem cell characters in glioma cells. To assess the function of glioma-derived extrinsic ribosome incorporation, we obtained ribosomes from the glioma cell line (U251MG) [26], and evaluated their effect on RICCS formation. Consistent with the findings in Figure 1 and Figure 2, glioma-derived extrinsic ribosome incorporation indeed promoted the cancer cell spheroids’ (GBM-RICCS) formation (Figure 3A). The sphere formation assay clearly showed that the numbers of spheroids in the GBM-RICCS formation were significantly increased, compared with the control sample (Figure 3B). In addition, stem cell markers, such as, SOX2 and Nestin, were also upregulated in GBM-RICCS compared with the control group (Figure 3C), suggesting that GBM-RICCS exhibits stem-like characters in glioma cells. Moreover, in accordance with the results using prokaryotic ribosome, GBM-RICCS formation by the glioma-derived extrinsic ribosome was markedly suppressed by RPS6 kinase inhibitor (PF47018671) (Figure 3D). In addition, PF4708671 treatment indeed downregulated SOX2 expression induced by RICCS (Supplementary Figure S3). No spheroid formation (>50µm) was observed in the PF47018671-treated group (data not shown). Those results indicated that not only prokaryotic ribosome, but also glioma-derived extrinsic ribosome promoted GBM-RICCS formation in glioma, and might suggest the potential biological roles of extrinsic ribosomal proteins through intrinsic RPS6 phosphorylation.

### 3.4. RPS6 Phosphorylation in Glioma Tissues

**Figure 4:**
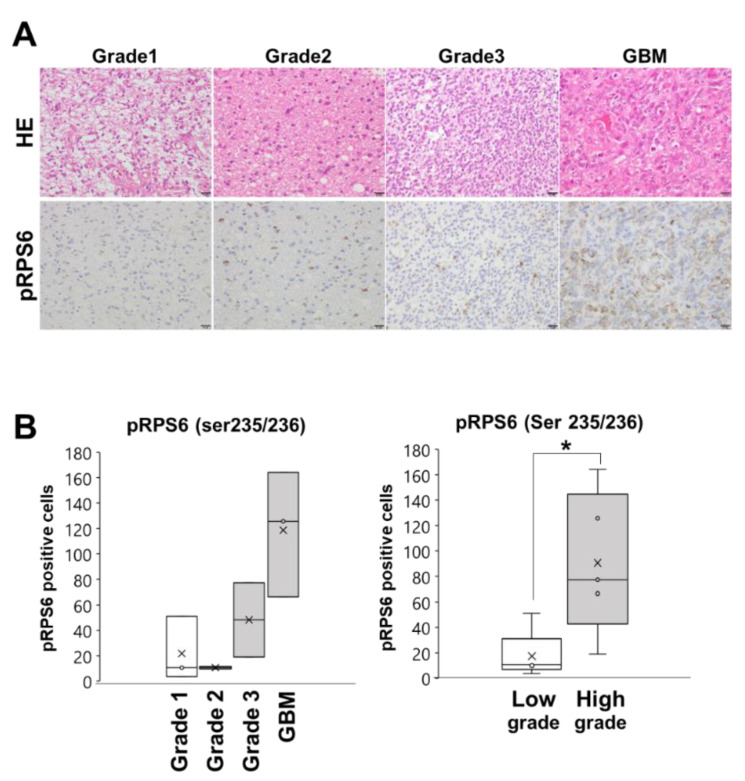
RPS6 phosphorylation in glioma tissues. (**A**), Representative images of hematoxylin and eosin (HE) staining (upper panel) and immunohistochemical staining for pRPS6 expression (lower panel) in human glioma tissues Grade 1, 2, 3, and 4 (GBM). Bar: 20 µm, (**B**), A graph showing the positive cells of pRPS6 in each grade of glioma patients (values are presented as mean ± SD of triplicate samples. * *p* < 0.05).

Previous studies indicated that, in GSC niches, ribosomal proteins play crucial roles in the development and maintenance of GSCs and are clinically associated with chemo-radio-resistance and GBM recurrence [3,4]. In addition, our previous results also revealed that, of these ribosomal proteins, RPS6 expression was upregulated in high malignant glioma patients [15]. Since our present results suggested that RPS6 phosphorylation might play important roles in glioma-derived extrinsic ribosome-induced GBM-RICCS formation (Figure 3), we next assessed RPS6 phosphorylation (ser235/236) in glioma patient tissues. Immunohistochemical staining analysis of RPS6 phosphorylation in glioma patient tissues clearly showed that RPS6 phosphorylation (pRPS6) was more strongly detected in high-grade glioma (grade. 3, GBM) compared with low-grade glioma patients (grade 1, 2) (Figure 4A). Moreover, as shown in Figure 4B, the number of pRPS6-positive cells in glioma patient tissues was markedly increased as the stage progressed and significantly high in higher grade glioma patients.

### 3.5. Possible Association between RPS6 Phosphorylation and GSC in Glioma Tissues

**Figure 5:**
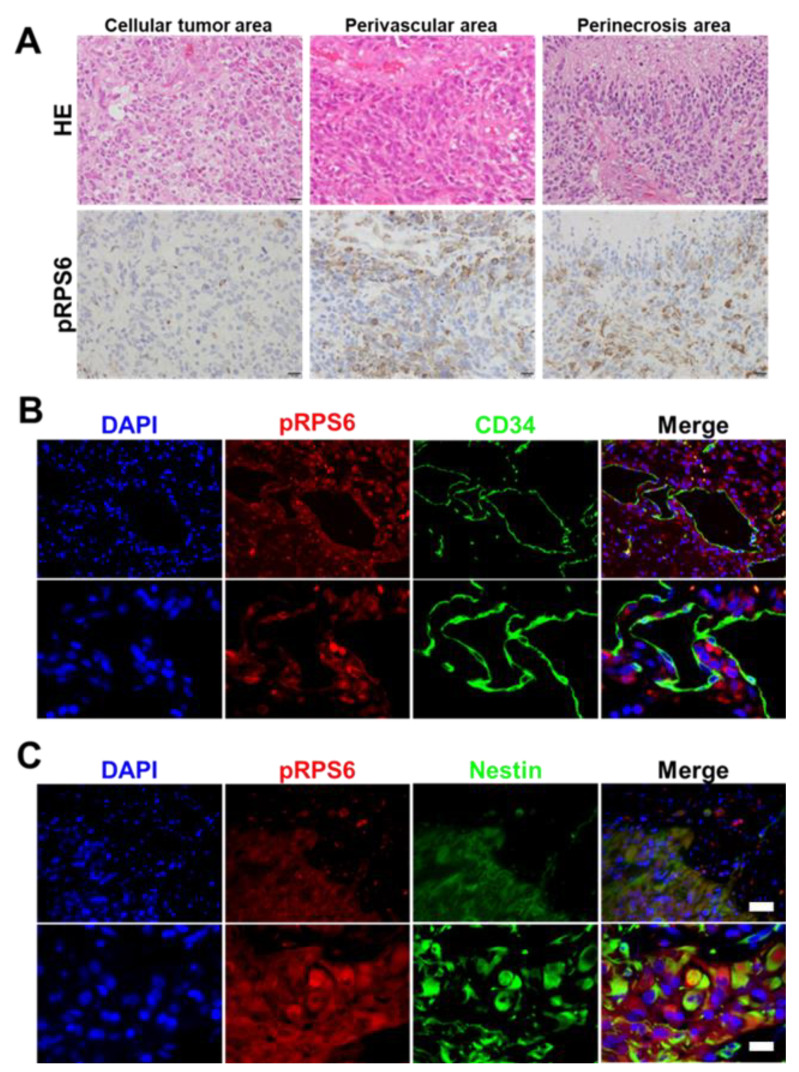
Possible association between RPS6 phosphorylation and GSC in glioma tissues. (**A**), Images showing higher expression of pRPS6 detected in the perivascular and perinecrosis area compared with the cellular tumor area. Bar: 20 µm. (**B**,**C**), Images showing double staining with pRPS6, CD34, and Nestin. Bar: 500 µm (each down-scale picture), 50 µm (up-scale picture).

In general, the peri-vascular niche and necrosis niche are critical for the growth of cancer stem cells, and chemo-radio resistance contributes to the short survival of brain tumors [27]. To further examine the possible roles of RPS6 phosphorylation induced by glioma-derived extrinsic ribosome incorporation in GSC development, we next assessed the detailed expression patterns of RPS6 phosphorylation (Ser235/236) at different characteristic sites in GBM tissues, especially the peri-vascular area. Vascular endothelial cells provide supportive environment factors for GSC growth, maintenance, and survival [7]. As shown in Figure 5A, RPS6 phosphorylation (Ser235/236) was predominantly expressed in the peri-necrosis area (middle panel) and peri-vascular area (right panel), compared with the cellular tumor area (left panel). Moreover, to evaluate pRPS6 in the perivascular niche, we assessed co-localization by evaluating the expression of CD34, a vascular endothelial marker (perivascular niche marker), by using double immune histochemistry staining. As shown in Figure 5B, pRPS6 frequently existed in the adjacent area of CD34-positive cells (double-positive cell rate: 3.95 ± 2.22%, Supplementary Table S1), suggesting that pRPS6 is predominantly expressed in the GSCs niche (peri vascular niche). We finally assessed co-localization by evaluating the expression of pRPS6 and Nestin (GSC marker) by using immunofluorescent double staining of GBM tissues. In contrast to the result shown in Figure 5B, almost all pRPS6-positive cells co-expressed Nestin (Figure 5C, double-positive cell rate: 73.15 ± 9.41%, Supplementary Table S1), indicating that pRPS6 expression may be associated with malignant progression through the development of GSC in GBM tissues.

## 4. Discussion

Ribosomal proteins are well-known to have functions of protein synthesis for homeostasis [9]. Recent studies have indicated that ribosomal proteins may play important roles not only in protein synthesis, but also in several other multi-functions, such as cell migration, glucose metabolism, and cell size [11,28]. Moreover, ribosome overexpression in several cancers, such as gastric cancer and hepatocellular cancer [28,29], may serve as a prognostic factor in tumor malignancy. Of these multiple functions, it is noteworthy that recent several studies have revealed that ribosomal protein may have the potential to play key roles in reprogramming and the development of stem cell characters. Ito et al. investigated and first reported that ribosome incorporation into somatic cells triggered stem cell characters [14,30]. Further studies also evaluated and revealed RICCS towards the EMT phenomenon and cell cluster formation in lung cancer and breast cancer [31,32], suggesting that ribosome incorporation may show physiological relevance and exhibit biological and clinical significance in the molecular mechanisms underlying the pathogenesis of tumor malignancy. In the present study, our results suggested that ribosome incorporation indeed induced stem cell characters in glioma cells. In addition, the glioma-derived extrinsic ribosome promoted GBM-RICCS formation in glioma, suggesting potential biological roles of extrinsic ribosomal proteins through intrinsic RPS6 phosphorylation. Moreover, in the clinic, pRPS6 expression may be associated with malignant progression through the development of GSCs in GBM tissues.

GSCs are highly associated with malignant phenomena, such as chemo-radio resistance and recurrence after operation [33]. Although elucidation of the molecular mechanism underlying GSC development is urgently needed, the relationship between GSC development and the key factor and trigger for reprogramming is totally unknown. Our data suggest that the glioma-derived extrinsic ribosome may serve as a key trigger for GSCs development through intrinsic RPS6 phosphorylation (Figure 1, Figure 2 and Figure 3). It has been reported that GSCs are dominantly expressed in niches, including the necrosis and border niche [8,34,35]. Our recent studies also revealed that ribosomal proteins are overexpressed at niches in GBM tissues [15]. In general, these niches contain quite stressful conditions associated with inflammation and nutrition and oxygen deficiency, suggesting that these areas may be exposed by GBM cellular contents (including ribosomal proteins) released by necrosis [27]. Furthermore, recent reports also provided evidence that GBM cells reportedly contain micro vesicles containing RNA and proteins that can communicate intracellularly to promote some malignant phenomena, including tumor growth [25]. It should be noted that pRPS6 expression was upregulated in these niches of glioma tissue specimens (Figure 5A–C). Taken together, these data suggest that extrinsic ribosome may influence pRPS6 expression via incorporation into GBM cells. Since the detailed molecular mechanism and clinical significance is still largely unknown, further investigation focusing on the relationship between extrinsic ribosome and GSC development is required.

Among the multiple functions of ribosomal proteins, it is notable that several recent studies have revealed that ribosome incorporation into somatic cells may have potential to play key roles in reprogramming and stem cell properties [29]. Here, we first found that ribosome-induced cancer cell spheroid (RICCS) formation was significantly upregulated by ribosome incorporation. As shown in Figure 1A–D, RICCS exhibited stem-like cell characters (number of cell spheroid, stem cell markers, and ability to transdifferentiate towards adipocytes and osteocytes). In RICCS, phosphorylation and protein expression of RPS6, an intrinsic ribosomal protein, and STAT3 phosphorylation were upregulated (Figure 2A), and might have regulated cell spheroid formation (Figure 2B). Moreover, consistently, glioma-derived extrinsic ribosome also promoted GBM-RICCS formation through intrinsic RPS6 phosphorylation (Figure 3). These results are the first reports showing the novel roles of ribosome incorporation in the development of GSCs, and suggest a new aspect of ribosome protein. Based on the results showing the overexpression of pRPS6 in highly malignant patients and predominantly expressed in GSC niches (Figure 4 and Figure 5), we additionally investigated the expression of ribosomal protein S6 kinase (RPS6K), an upstream kinase phosphorylating RPS6, by IVY GAP (glioblastoma atlas project) database (http://glioblastoma.alleninstitute.org/, accessed on 1 November 2021). Several RPS6Ks were upregulated in the predominantly existing GSC area (data not shown). Furthermore, since RPS6 phosphorylation was also upregulated not only in GBM-RICCS, but also in clinical GBM tissue specimens, these data suggested that ribosome incorporation might phosphorylate RPS6 through the direct or indirect activation of RPS6Ks. Meanwhile, GBM cells reportedly promote micro vesicles containing RNA, proteins that regulate GBM malignant phenomena including tumor growth and developing GSCs [25,36]. A previous study reported that nucleolar regulation promotes GBM malignancy via the production of ribosomal proteins [37,38]. Therefore, ribosomes are reported to represent inflammation-inducing ribosome biogenesis [39], such as the perinecrosis niche and perivascular niche. These findings suggested GBM cells potentially induced stem cell characters not only by intrinsic ribosome but also extrinsic ribosome. Further elucidation of the detailed molecular mechanisms focusing on both the intrinsic and extrinsic ribosome will contribute to the identification of new strategies of mechanisms upregulating GSCs in niches and RPS6 phosphorylation will be a potential target for GBM patients. It has been reported that RPS6 phosphorylation regulates malignant phenotypes in several cancers induced by Akt-mTOR signaling [19]. Several cancers are associated with these signals by autophagy via the incorporation of micro vesicles, such as RNA, DNA, and proteins [36]. In addition, a previous study revealed that RICCS induced the autophagy phenomenon in breast cancer cells [31]. These findings indicate that RICCS may induce RPS6 phosphorylation via autophagy, such as virus and bacteria. In addition, our preliminary results showed that RPS6 knockdown suppressed phospho-JAK2, and the sphere-forming ability induced by IL-6, a STAT3-activating ligand, was abrogated by siRPS6. These findings suggest that RPS6 phosphorylation may regulate the upstream area of STAT3, such as IL-6R or IL-6 levels. In the future study, we will focus on and further evaluate the involvement of RPS6 phosphorylation in the molecular mechanisms underlying GBM-RICCS formation.

## 5. Conclusions

The present study first revealed that glioma cells acquire stem-like characters by extrinsic ribosome stimuli. The glioma-derived extrinsic ribosome incorporation promoted GBM-RICCS formation in glioma through intrinsic RPS6 phosphorylation. Finally, RPS6 phosphorylation may be associated with malignant progression through the development of GSCs in GBM tissues. Further investigation focusing on the biological and clinical significance of ribosome incorporation may contribute to elucidation of the detailed molecular pathogenesis of GSC development, and open up new insights into improving the treatment of highly malignant GBM patients.

## Data Availability

Not applicable.

## Supplementary Materials

The following are available online at https://www.mdpi.com/article/10.3390/cells10112970/s1, Figure S1: Representative pictures of immunohistochemical analysis for RICCS, Figure S2: Western blotting showing the stem cell markers, Nestin in U251MG cells, Figure S3: Western blotting showing the stem cell markers, Sox2 in RICCS, Table S1: Co-localization of phosphorylated RPS6 with Nestin or CD34 in glioma tissues.

## References

[1] Louis D.N., Ohgaki H., Wiestler O.D., Cavenee W.K., Burger P.C., Jouvet A., Scheithauer B.W., Kleihues P. (2007). The 2007 WHO Classification of Tumours of the Central Nervous System. Acta Neuropathol..

[2] Stupp R., Mason W.P., Bent M.V.D., Weller M., Fisher B., Taphoorn M.J., Belanger K., Brandes A., Marosi C., Bogdahn U. (2005). Radiotherapy plus Concomitant and Adjuvant Temozolomide for Glioblastoma. N. Engl. J. Med..

[3] Beier D., Schulz J.B., Beier C.P. (2011). Chemoresistance of glioblastoma cancer stem cells—Much more complex than expected. Mol. Cancer.

[4] Vescovi A.L., Galli R., Reynolds B.A. (2006). Brain tumour stem cells. Nat. Rev. Cancer.

[5] Singh S.K., Hawkins C., Clarke I.D., Squire J., Bayani J., Hide T., Henkelman R.M., Cusimano M., Dirks P.B. (2004). Identification of human brain tumour initiating cells. Nature.

[6] Biserova K., Jakovlevs A., Uljanovs R., Strumfa I. (2021). Cancer Stem Cells: Significance in Origin, Pathogenesis and Treatment of Glioblastoma. Cells.

[7] Calabrese C., Poppleton H., Kocak M., Hogg T.L., Fuller C., Hamner B., Oh E.Y., Gaber M., Finklestein D., Allen M. (2007). A Perivascular Niche for Brain Tumor Stem Cells. Cancer Cell.

[8] Seidel S., Garvalov B.K., Wirta V., Von Stechow L., Schänzer A., Meletis K., Wolter M., Sommerlad D., Henze A.-T., Nistér M. (2010). A hypoxic niche regulates glioblastoma stem cells through hypoxia inducible factor 2α. Brain.

[9] Kapp L.D., Lorsch J.R. (2004). The Molecular Mechanics of Eukaryotic Translation. Annu. Rev. Biochem..

[10] Klinge S., Voigts-Hoffmann F., Leibundgut M., Ban N. (2012). Atomic structures of the eukaryotic ribosome. Trends Biochem. Sci..

[11] Zhou X., Liao W.-J., Liao J.-M., Liao P., Lu H. (2015). Ribosomal proteins: Functions beyond the ribosome. J. Mol. Cell Biol..

[12] Khatter H., Myasnikov A.G., Natchiar S.K., Klaholz B.P. (2015). Structure of the human 80S ribosome. Nature.

[13] Ito N., Anam M.B., Ahmad S.A.I., Ohta K. (2018). Transdifferentiation of human somatic cells by ribosome. Dev. Growth Differ..

[14] Ito N., Katoh K., Kushige H., Saito Y., Umemoto T., Matsuzaki Y., Kiyonari H., Kobayashi D., Soga M., Era T. (2018). Ribosome Incorporation into Somatic Cells Promotes Lineage Transdifferentiation towards Multipotency. Sci. Rep..

[15] Shirakawa Y., Hide T., Yamaoka M., Ito Y., Ito N., Ohta K., Shinojima N., Mukasa A., Saito H., Jono H. (2020). Ribosomal protein S6 promotes stem-like characters in glioma cells. Cancer Sci..

[16] Sojka L., Fučík V., Krásný L., Barvík I., Jonak J. (2007). YbxF, a Protein Associated with Exponential-Phase Ribosomes in Bacillus subtilis. J. Bacteriol..

[17] Han Z., Zhang Q., Zhu Y., Chen J., Li W. (2020). Ribosomes: An Exciting Avenue in Stem Cell Research. Stem Cells Int..

[18] Khalaileh A., Dreazen A., Khatib A., Apel R., Swisa A., Kidess-Bassir N., Maitra A., Meyuhas O., Dor Y., Zamir G. (2013). Phosphorylation of Ribosomal Protein S6 Attenuates DNA Damage and Tumor Suppression during Development of Pancreatic Cancer. Cancer Res..

[19] Chen B., Tan Z., Gao J., Wu W., Liu L., Jin W., Cao Y., Zhao S., Zhang W., Qiu Z. (2015). Hyperphosphorylation of ribosomal protein S6 predicts unfavorable clinical survival in non-small cell lung cancer. J. Exp. Clin. Cancer Res..

[20] Pallis M., Harvey T., Russell N. (2016). Phenotypically Dormant and Immature Leukaemia Cells Display Increased Ribosomal Protein S6 Phosphorylation. PLoS ONE.

[21] West A.J., Tsui V., Stylli S.S., Nguyen H.P.T., Morokoff A.P., Kaye A.H., Luwor R.B. (2018). The role of interleukin-6-STAT3 signalling in glioblastoma. Oncol. Lett..

[22] Leidgens V., Proske J., Rauer L., Moeckel S., Renner K., Bogdahn U., Riemenschneider M.J., Proescholdt M., Vollmann-Zwerenz A., Hau P. (2017). Stattic and metformin inhibit brain tumor initiating cells by reducing STAT3-phosphorylation. Oncotarget.

[23] Piperi C., Papavassiliou K.A., Papavassiliou A.G. (2019). Pivotal Role of STAT3 in Shaping Glioblastoma Immune Microenvironment. Cells.

[24] Birner P., Toumangelova-Uzeir K., Natchev S., Guentchev M. (2010). STAT3 tyrosine phosphorylation influences survival in glioblastoma. J. Neuro-Oncol..

[25] Skog J., Wurdinger T., van Rijn S., Meijer D.H., Gainche L., Curry W.T., Carter B.S., Krichevsky A.M., Breakefield X.O. (2008). Glioblastoma microvesicles transport RNA and protein that promote tumor growth and provide diagnostic biomarkers Johan. Nat. Cell Biol..

[26] Anger A.M., Armache J.-P., Berninghausen O., Habeck M., Subklewe M., Wilson D., Beckmann R. (2013). Structures of the human and Drosophila 80S ribosome. Nature.

[27] Hambardzumyan D., Bergers G. (2015). Glioblastoma: Defining Tumor Niches. Trends Cancer.

[28] Catez F., Venezia N.D., Marcel V., Zorbas C., Lafontaine D.L., Diaz J.-J. (2019). Ribosome biogenesis: An emerging druggable pathway for cancer therapeutics. Biochem. Pharmacol..

[29] Brighenti E., Treré D., Derenzini M. (2015). Targeted cancer therapy with ribosome biogenesis inhibitors: A real possibility?. Oncotarget.

[30] Ito N., Ohta K. (2015). Reprogramming of human somatic cells by bacteria. Dev. Growth Differ..

[31] Kudo M., Anam M.B., Istiaq A., Ahmad S.A.I., Ito N., Ohta K. (2021). Ribosome Incorporation Induces EMT-like Phenomenon with Cell Cycle Arrest in Human Breast Cancer Cell. Cells Tissues Organs.

[32] Anam M.B., Istiaq A., Kariya R., Kudo M., Ahmad S.A.I., Ito N., Okada S., Ohta K. (2021). Ribosome induces transdifferentiation of A549 and H-111-TC cancer cell lines. Biochem. Biophys. Rep..

[33] Gimple R.C., Bhargava S., Dixit D., Rich J.N. (2019). Glioblastoma stem cells: Lessons from the tumor hierarchy in a lethal cancer. Genes Dev..

[34] Ishii A., Kimura T., Sadahiro H., Kawano H., Takubo K., Suzuki M., Ikeda E. (2016). Histological Characterization of the Tumorigenic “Peri-Necrotic Niche” Harboring Quiescent Stem-Like Tumor Cells in Glioblastoma. PLoS ONE.

[35] Hide T., Komohara Y., Miyasato Y., Nakamura H., Makino K., Takeya M., Kuratsu J.-I., Mukasa A., Yano S. (2018). Oligodendrocyte Progenitor Cells and Macrophages/Microglia Produce Glioma Stem Cell Niches at the Tumor Border. EBioMedicine.

[36] Wang J., Liu J., Sun G., Meng H., Guan Y., Yin Y., Zhao Z., Dong X., Yin S., Li H. (2019). Glioblastoma extracellular vesicles induce the tumour-promoting transformation of neural stem cells. Cancer Lett..

[37] Kofuji S., Hirayama A., Eberhardt A.O., Kawaguchi R., Sugiura Y., Sampetrean O., Ikeda Y., Warren M., Sakamoto N., Kitahara S. (2019). IMP dehydrogenase-2 drives aberrant nucleolar activity and promotes tumorigenesis in glioblastoma. Nature.

[38] Derenzini M., Trerè D., Pession A., Govoni M., Sirri V., Chieco P. (2000). Nucleolar size indicates the rapidity of cell proliferation in cancer tissues. J. Pathol..

[39] Brighenti E., Giannone F.A., Fornari F., Onofrillo C., Govoni M., Montanaro L., Treré D., Derenzini M. (2016). Therapeutic dosages of aspirin counteract the IL-6 induced pro-tumorigenic effects by slowing down the ribosome biogenesis rate. Oncotarget.

