# Supplementary material for: Glioma Cells Acquire Stem-like Characters by Extrinsic Ribosome Stimuli
Source: Cells. 2021 Nov 1;10(11):2970. doi: 10.3390/cells10112970 (PMC8616507; doi:10.3390/cells10112970)
Supplement: Supplementary file 1 [file cells-10-02970-s001.zip › cells-1400506-supplementary.pdf]

cells

Supplementary Material

Glioma Cells Acquire Stem-like Characters by Extrinsic Ribosome Stimuli

Yuki Shirakawa [1], Kunimasa Ohta [2], Shunsuke Miyake [1,3], Ayumi Kanemaru [1], Akari Kuwano [1], Kou Yonemaru [1], Shota Uchino [1], Michiko Yamaoka [1], Yuki Ito [1], Naofumi Ito [4], Takuichiro Hide [5], Naoki Shinojima [6], Akitake Mukasa [6], Hideyuki Saito [1,3] and Hirofumi Jono [1,3,*]

1 Department of Clinical Pharmaceutical Sciences, Graduate School of Pharmaceutical Sciences, Kumamoto University, Kumamoto 860-8556, Japan; y.shirakawa.da@juntendo.ac.jp (Y.S.); 191y2005@st.kumamoto-u.ac.jp (S.M.); 197y2002@st.kumamoto-u.ac.jp (A.K.); ak.1212@icloud.com (A.K.); 179p1054@st.kumamoto-u.ac.jp (K.Y.); 175p1008@st.kumamoto-u.ac.jp (S.U.); yfe31628@gmail.com (M.Y.); 161p1003@st.kumamoto-u.ac.jp (Y.I.); saitohide@kuh.kumamoto-u.ac.jp (H.S.)

2 Department of Stem Cell Biology, Faculty of Arts and Science, Kyushu University, Fukuoka 819-0385, Japan; ohta9203@artsci.kyushu-u.ac.jp

3 Department of Pharmacy, Kumamoto University Hospital, Kumamoto 860-8556, Japan

4 Department of Developmental Neurobiology, Graduate School of Life Sciences, Kumamoto University, Kumamoto 860-8556, Japan; mikan03orange@gmail.com

5 Department of Neurosurgery, Kitasato University School of Medicine, 252-0375, Kanagawa, Japan; thide@med.kitasato-u.ac.jp

6 Department of Neurosurgery, Graduate School of Medical Sciences, Kumamoto University, Kumamoto 860-8556, Japan; nshinojima-kuh@umin.ac.jp (N.S.); mukasa@kumamoto-u.ac.jp (A.M.)

* Correspondence: hjono@kuh.kumamoto-u.ac.jp; Tel.: +81-96-373-5823

Table S1. Co-localization of phosphorylated RPS6 with Nestin or CD34 in glioma tissues.

Ave. double-positive cells rate / image (%)	
Nestin + /pRPS6 +	73.15 ± 9.41
CD34 + /pRPS6 +	3.95 ± 2.22

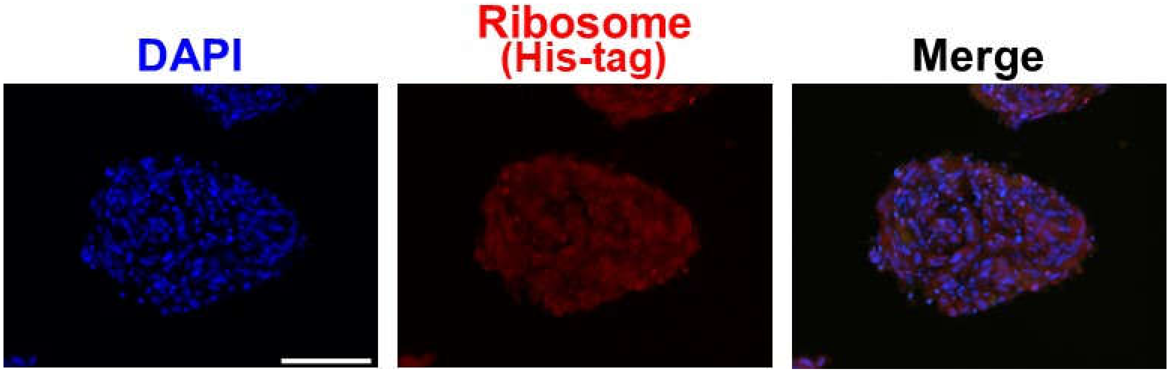

Figure S1. Representative pictures of immunohistochemical analysis for RICCS. Images showing immunohistochemical staining for His-tag conjugated ribosome in RICCS.

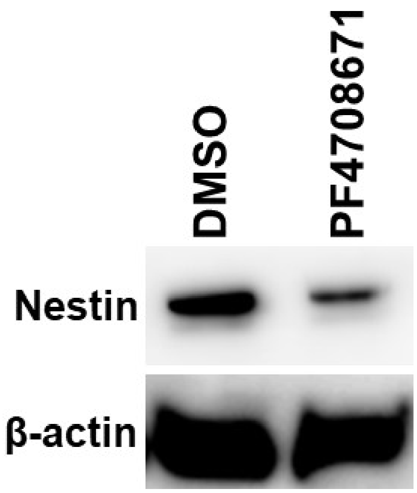

Figure S2. Western blotting showing the stem cell markers, Nestin in U251MG cells. PF4708671 reduced Nestin expression in U251MG cells.

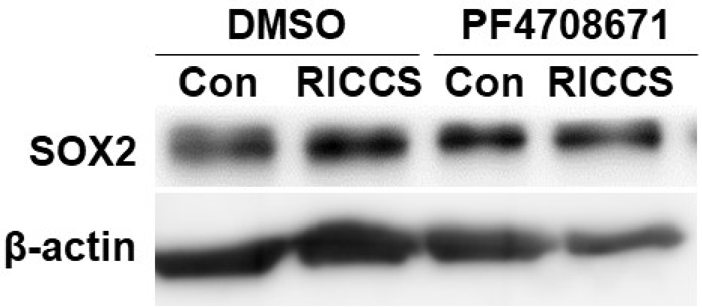

Figure S3. Western blotting showing the stem cell markers, Sox2 in RICCS. Sox2 expression in RICCS was down-regulated by PF4708671 treatment.
